# Supplementary material for: Modeling the Role of Relationship Fading and Breakup in Social Network Formation
Source: PLoS One. 2015 Jul 15;10(7):e0133005. doi: 10.1371/journal.pone.0133005 (PMC4503738; doi:10.1371/journal.pone.0133005)
Supplement: S1 Appendix — (PDF) [file pone.0133005.s001.pdf]

Linear stability analysis

In this Appendix, a linear stability analysis of the link weight w around the equilibrium value $\langle w \rangle$ for the aging model is demonstrated. In order to evaluate the linear stability of w around $\langle w \rangle$, we consider the case that one of the links, say the link between nodes i and j, is stronger than other links by Δw while other links have the same weight as $\langle w \rangle$. The expected amount of reinforcement on the link ij in one time step, $\langle w_+ \rangle$, is given by

$$\begin{aligned} \langle w_+ \rangle = {} & 2 \cdot \frac{w + \Delta w}{w(n-1) + \Delta w} \\ & + (n-2) \cdot \frac{2}{n-1} \cdot \frac{w + \Delta w}{(n-2)w + \Delta w} \\ & + 2 \cdot \frac{(n-2)w}{w(n-1) + \Delta w} \cdot \frac{1}{n-2}. \end{aligned} \tag{1}$$

The first, second, and third terms correspond to the case that the link ij is selected as the first, second, and third link in one LA event, respectively. LA must start from the node i or j when the link ij is selected either as the first or the third link, which gives 2 in the first and the third terms. When the link ij is selected as the second link, the LA event must start from the node except for i and j, which yields the factor $n-2$ in the second term. If we assume $\Delta w \ll 1$ and ignore the higher order terms of Δw, the expected increase $\langle w_+ \rangle$ is calculated as

$$\langle w_+ \rangle \approx \frac{6}{n-1} + \frac{2\Delta w}{w(n-1)} \left(2 - \frac{2}{n-1} + \frac{1}{n-2} \right). \tag{2}$$

On the other hand, the expected amount of the decrease due to aging, $\langle w_- \rangle$, is $(w + \Delta w)(1-f)$. When $w = \langle w \rangle$, the total change of the link weight is calculated as

$$\langle w_+ \rangle - \langle w_- \rangle = \Delta w (1-f) \cdot \frac{-n^2 + 3}{3(n-1)(n-2)}, \tag{3}$$

which always has a negative sign of Δw for $n \geq 3$, indicating the dynamics of Δw is linearly stable around its equilibrium value $\langle w \rangle$. This demonstrates that the link weight within communities gets closer to its equilibrium point with time. Since the equilibrium link weight is shared in a community, the link weights in a community become homogeneous.
